# Geomfinder: a multi-feature identifier of similar three-dimensional protein patterns: a ligand-independent approach

**DOI:** 10.1186/s13321-016-0131-9

**Published:** 2016-04-18

**Authors:** Gabriel Núñez-Vivanco, Alejandro Valdés-Jiménez, Felipe Besoaín, Miguel Reyes-Parada

**Affiliations:** Escuela de Ingeniería Civil en Bioinformática, Universidad de Talca, Avenida Lircay s/n, Talca, Chile; Centro de Bioinformática y Simulación Molecular, Universidad de Talca, 2 Norte 685, Talca, Chile; School of Medicine, Faculty of Medical Sciences, Universidad de Santiago de Chile, Avenida Libertador Bernardo O’Higgins 3363, Santiago, Chile; Facultad de Ciencias de la Salud, Universidad Autonóma de Chile, 5 Poniente 1670, Talca, Chile; Estudis d’Informática, Multimedia i Telecomunicacio, Universitat Oberta de Catalunya, Rambla del Poblenou 15, Barcelona, Spain; Internet Interdisciplinary Institute (IN3), Universitat Oberta de Catalunya, Av. Carl Friedrich Gauss, 5, Castelldefels, Barcelona, Spain

**Keywords:** Pattern, Similarity, Protein-structure

## Abstract

**Background:**

Since the structure of proteins is more conserved than the sequence, the identification of conserved three-dimensional (3D) patterns among a set of proteins, can be important for protein function prediction, protein clustering, drug discovery and the establishment of evolutionary relationships. Thus, several computational applications to identify, describe and compare 3D patterns (or motifs) have been developed. Often, these tools consider a 3D pattern as that described by the residues surrounding co-crystallized/docked ligands available from X-ray crystal structures or homology models. Nevertheless, many of the protein structures stored in public databases do not provide information about the location and characteristics of ligand binding sites and/or other important 3D patterns such as allosteric sites, enzyme-cofactor interaction motifs, etc. This makes necessary the development of new ligand-independent methods to search and compare 3D patterns in all available protein structures.

**Results:**

Here we introduce Geomfinder, an intuitive, flexible, alignment-free and ligand-independent web server for detailed estimation of similarities between all pairs of 3D patterns detected in any two given protein structures. We used around 1100 protein structures to form pairs of proteins which were assessed with Geomfinder. In these analyses each protein was considered in only one pair (e.g. in a subset of 100 different proteins, 50 pairs of proteins can be defined). Thus: (a) Geomfinder detected identical pairs of 3D patterns in a series of monoamine oxidase-B structures, which corresponded to the effectively similar ligand binding sites at these proteins; (b) we identified structural similarities among pairs of protein structures which are targets of compounds such as acarbose, benzamidine, adenosine triphosphate and pyridoxal phosphate; these similar 3D patterns are not detected using sequence-based methods; (c) the detailed evaluation of three specific cases showed the versatility of Geomfinder, which was able to discriminate between similar and different 3D patterns related to binding sites of common substrates in a range of diverse proteins.

**Conclusions:**

Geomfinder allows detecting similar 3D patterns between any two pair of protein structures, regardless of the divergency among their amino acids sequences. Although the software is not intended for simultaneous multiple comparisons in a large number of proteins, it can be particularly useful in cases such as the structure-based design of multitarget drugs, where a detailed analysis of 3D patterns similarities between a few selected protein targets is essential.

**Electronic supplementary material:**

The online version of this article (doi:10.1186/s13321-016-0131-9) contains supplementary material, which is available to authorized users.

## Background

Current approaches for protein function prediction as well as for protein clustering and classification, are based on the use of both sequence and/or structural information [1, 2]. Nevertheless, considering that the structure of proteins is several times more conserved than their sequences [3], it is increasingly recognized that methods based on structural data can be more informative for the aforementioned purposes. In addition, the identification of conserved three-dimensional (3D) patterns among a set of proteins (related or not between them), could represent a key event on the structural convergent evolution of the queried proteins. Moreover, as in some cases these 3D patterns can be part of the binding/catalytic sites of the proteins, the identification of their characteristics and the assessment of their similarities can be useful for the development of new lead compounds and the rational design of polypharmacological drugs [2, 4–6]. It should be noted that in many cases functionally relevant structural motifs such as catalytic sites or ligand-binding sites occur only once in a protein structure. Nevertheless, a number of other important 3D patterns such as allosteric sites, protein-protein interaction motifs or ion binding sites might occur several times in a given protein. For instance, numerous allosteric sites have been identified in G protein-coupled receptors [7]. Indeed, computational mapping in muscarinic receptors has revealed the existence of up to seven allosteric sites [8]. A similar situation is observed in ligand-gated ion channels (e.g. nicotinic acethylcholine receptors; [9, 10]) which contain allosteric binding sites in their extracellular, transmembrane and intracellular domains. Likewise, protein-protein or lipid-protein interactions can be founded in the occurrence of numerous distinct interfaces in the interacting protein(s) [11, 12].

This background has motivated the development of several computational applications to identify, describe and compare 3D patterns (or motifs) (e.g [13–18]), some of which are specifically focused on protein ligand-binding sites (see [19–22] for reviews). Most of these approaches implicate the estimation of a scoring function, based on the comparison of geometric, energetic, sequence-based or chemical features of known motifs or binding sites. Thus, parameters such as the solvent-accessible area, Van der Waals and electrostatic energies and sequence similarity, have been widely used [23–29]. These approaches have proved to be useful for protein clustering, drug repurposing, protein classification, drug discovery and the establishment of evolutionary relationships [30–34].

Often, these methodologies consider 3D patterns as: (a) those described by the residues surrounding co-crystallized ligands/ions available from X-ray crystal structures, and b) those identified by sequence-based methods (e.g. PROSITE consensus patterns; [35]). Nevertheless, nearly 30 % of the protein structures stored in the Protein Data Bank (PDB)[36], do not provide information about the exact location of their ligand binding sites [37]. Indeed, even in those cases where these data exist (for both, sequence and structural patterns), they usually refer to the orthosteric site, but do not consider allosteric sites which have been shown to be fundamental for protein function and drug design [10, 38]. Additionally, more than 3 million of protein homology models have been deposited in public databases [39], and in many cases they neither offer data about the putative ligand-binding sites. This scenery makes necessary the development of new ligand-independent methods in order to allow in-depth assessment of unknown 3D patterns in all available protein structures.

Here we introduce Geomfinder, an intuitive, flexible, alignment-free and ligand-independent web server for an exhaustive searching of similarities among pairs of 3D patterns detected in two given protein structures (X-ray or homology models). Remarkably, our software works regardless of the previous existence of information about the presence of ligands/ions, motif and/or binding site characteristics at the investigated proteins.

## Implementation

**Fig. 1:**
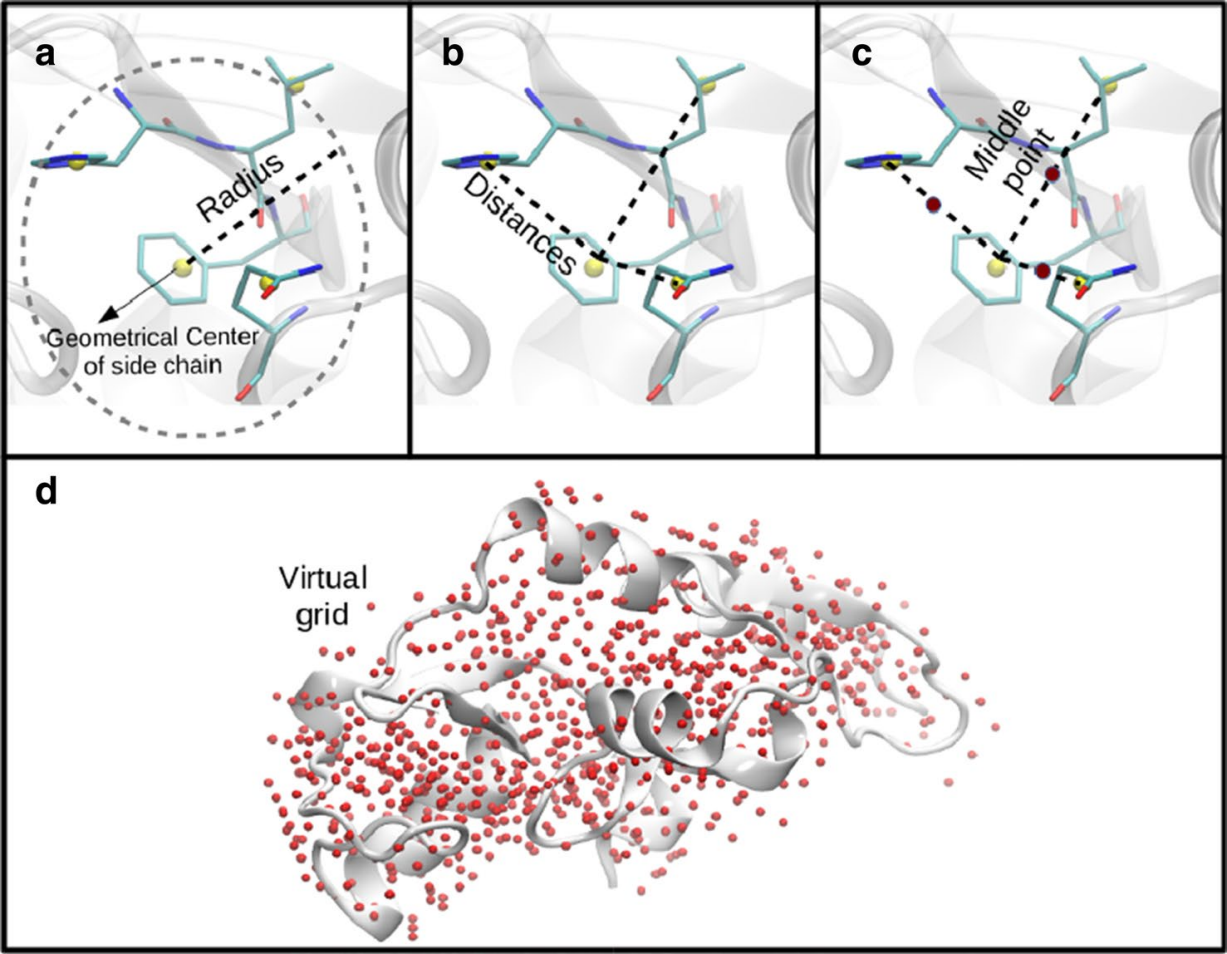
Virtual grid of reference coordinates. This figure shows the process to build a grid of reference coordinates

Geomfinder is a free access web-based application that estimates the similarity between all possible 3D patterns contained in any given pair of protein structures (e.g. protein A and protein B). These patterns are represented as a set of residues located at certain distances (defined by the user) between them. This application is composed of four main steps:

**The first step** generates on each protein a virtual grid of coordinates which represents the initial location to find the 3D patterns and it is constructed as follows:

- All coordinates of the geometrical center of the side chain of the residues located at a user-defined distance (radius) are selected (Fig. 1a).
- The distances between each pair of the previously selected coordinates are individually calculated (Fig. 1b).
- The middle point between all measured distances is calculated (Fig. 1c).
- A virtual grid is defined with all middle points coordinates (Fig. 1d).

In **the second step** all possible 3D patterns occurring in each of the proteins of interest are detected, using as reference the virtual grid already generated.

**The third step** generates a list of four descriptors for each 3D pattern identified (described in the next section).

**The fourth step** makes use of the descriptors previously estimated to calculate a single similarity score among any possible pair of 3D patterns found in each queried protein. This is done in an all-against-all procedure, and finally generates a list of pairs of 3D patterns, shown as interactive data tables and a Jmol viewer.

**Fig. 2:**
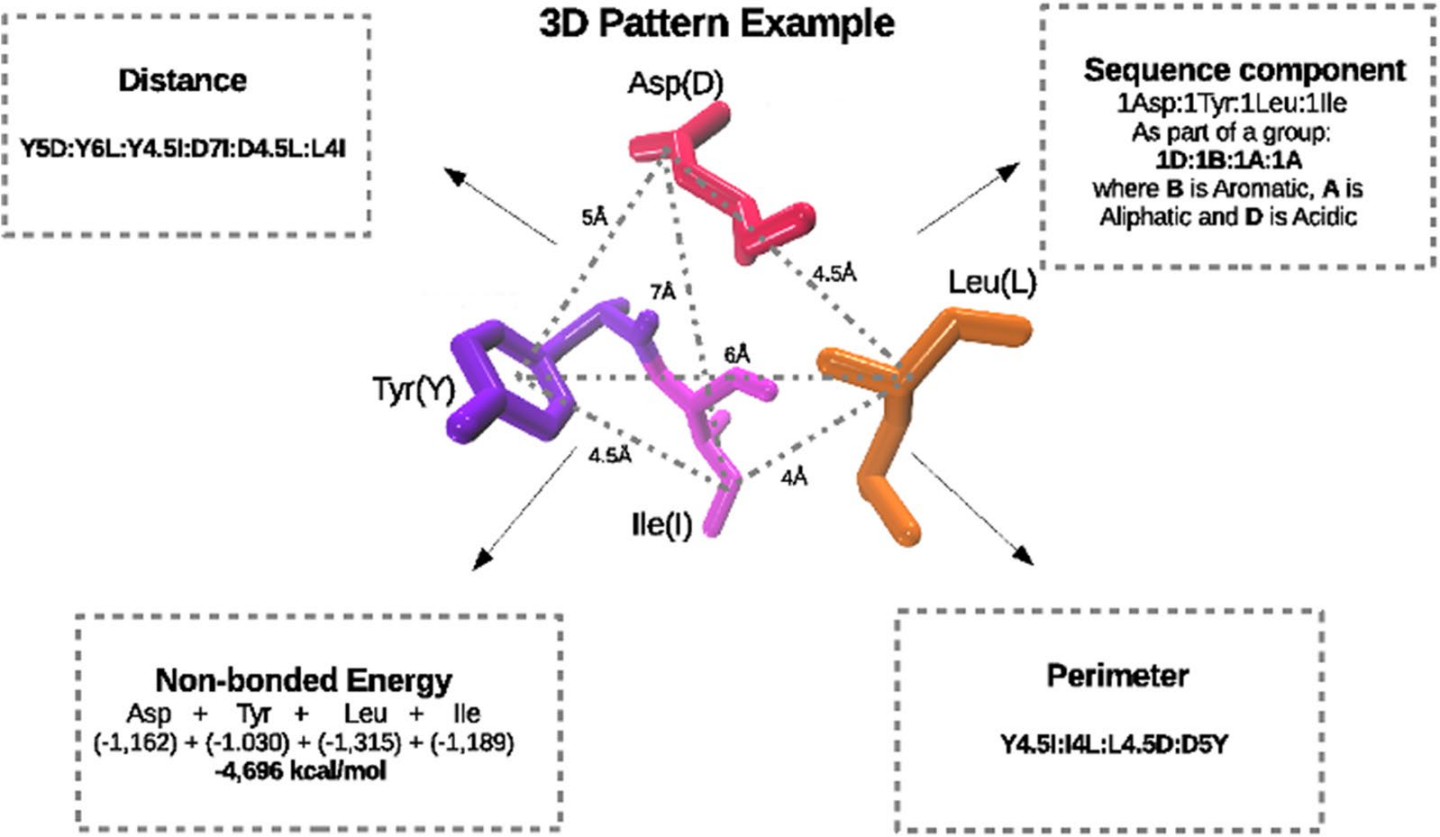
Four descriptors calculated from each 3D pattern. As an example, a 3D pattern is shown in licorice format. Different colors denotes different residues (*red* Asp, *orange* Leu, pink: *Ile* and *violet* Tyr). *Squares* in segmented lines, show each descriptor obtained from this example of 3D pattern

**Fig. 3:**
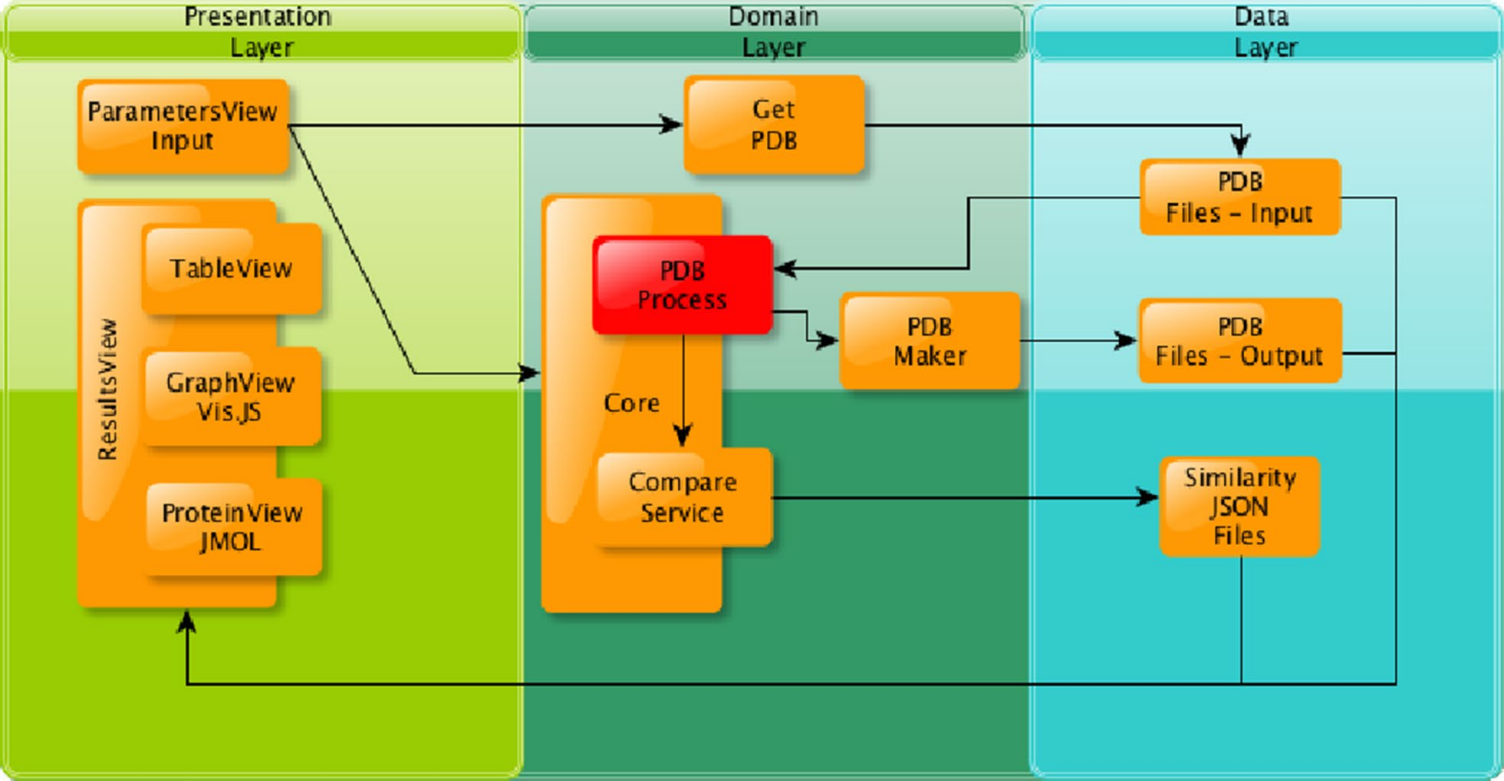
Implemented architecture and essential components and services. The architecture consists of three main layers: the presentation, domain and data layers, representing the interaction between the essential components of the solution

**Fig. 4:**
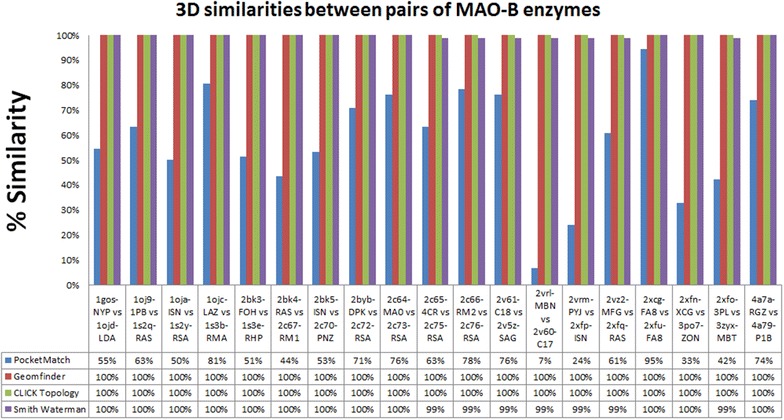
Similarity of the sequence and the 3D patterns in the Monoamine Oxidase B proteins. The *red bars* represent the highest value of a GScore between each pair of 3D patterns related with MAO-B binding sites. The sequence similarity between each pair of MAO proteins is shown in the *purple bars*. The *blue* and *green bars* show the similarity measured by the PocketMatch and the Click software, respectively. All pair of MAO proteins evaluated together with the % of similarity of all methods, are described in the X axis. The protein sequences were obtained with the babel software, using as input the structures obtained from the PDB database

**Fig. 5:**
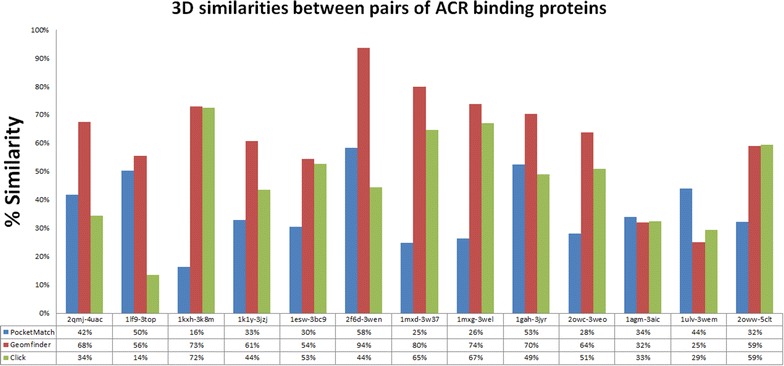
Similarity of the sequence and the 3D patterns in the ACR binding proteins. The *red bars* represent the highest value of a GScore between each pair of 3D patterns related with ACR binding sites. The *blue* and *green bars* show the similarity measured by the PocketMatch and the Click software, respectively. All pair of MAO proteins evaluated together with the % of similarity of all methods, are described in the X axis

**Fig. 6:**
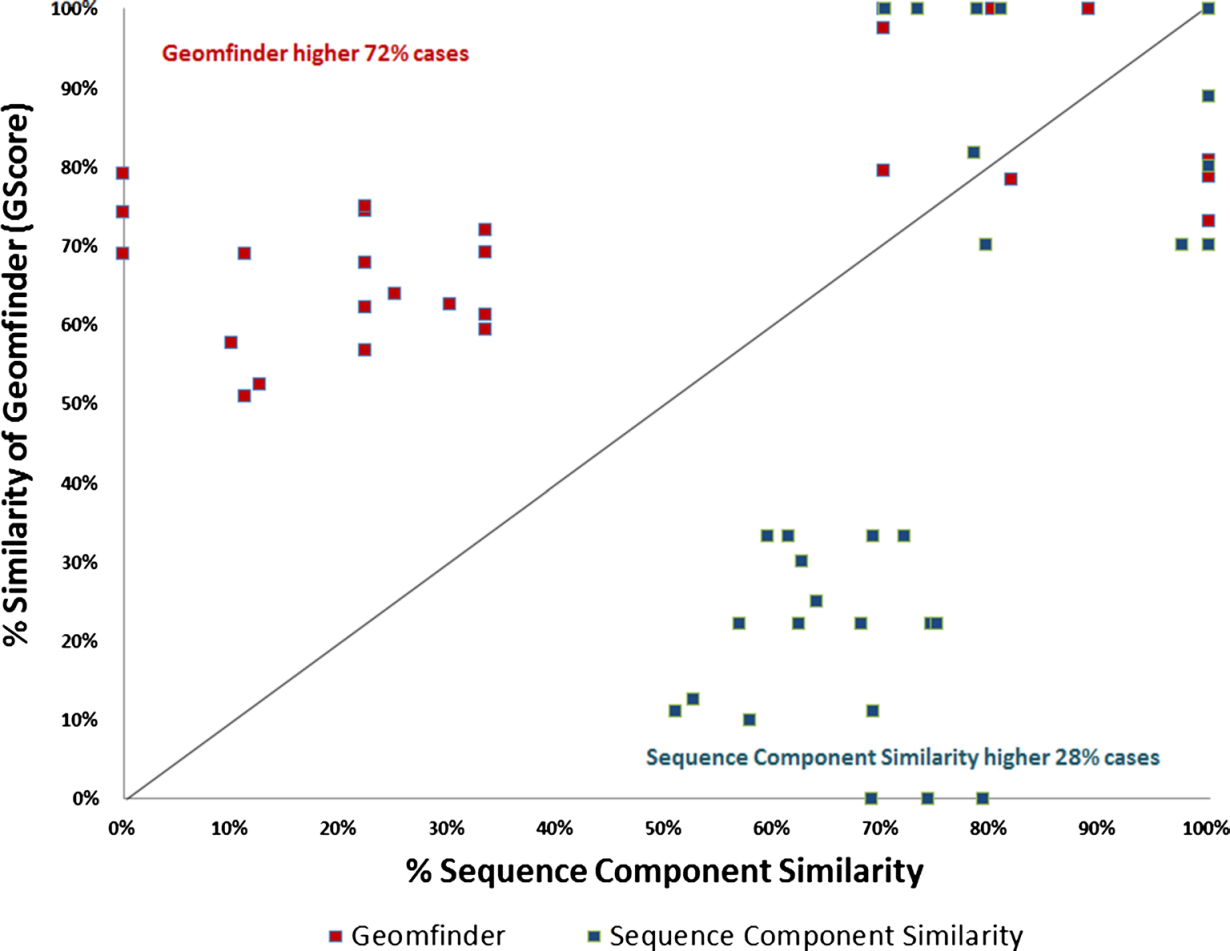
Similarity of the 3D patterns in the Benzamidine (BEN) binding proteins. In *red* is represented the higher value of the GScore (Geomfinder) between each pair of 3D patterns related with the BEN binding sites. The Sequence Component Similarity between each pair of BEN target proteins is shown in *blue*. All pair of the evaluated proteins are described in this* scatter plot*

**Fig. 7:**
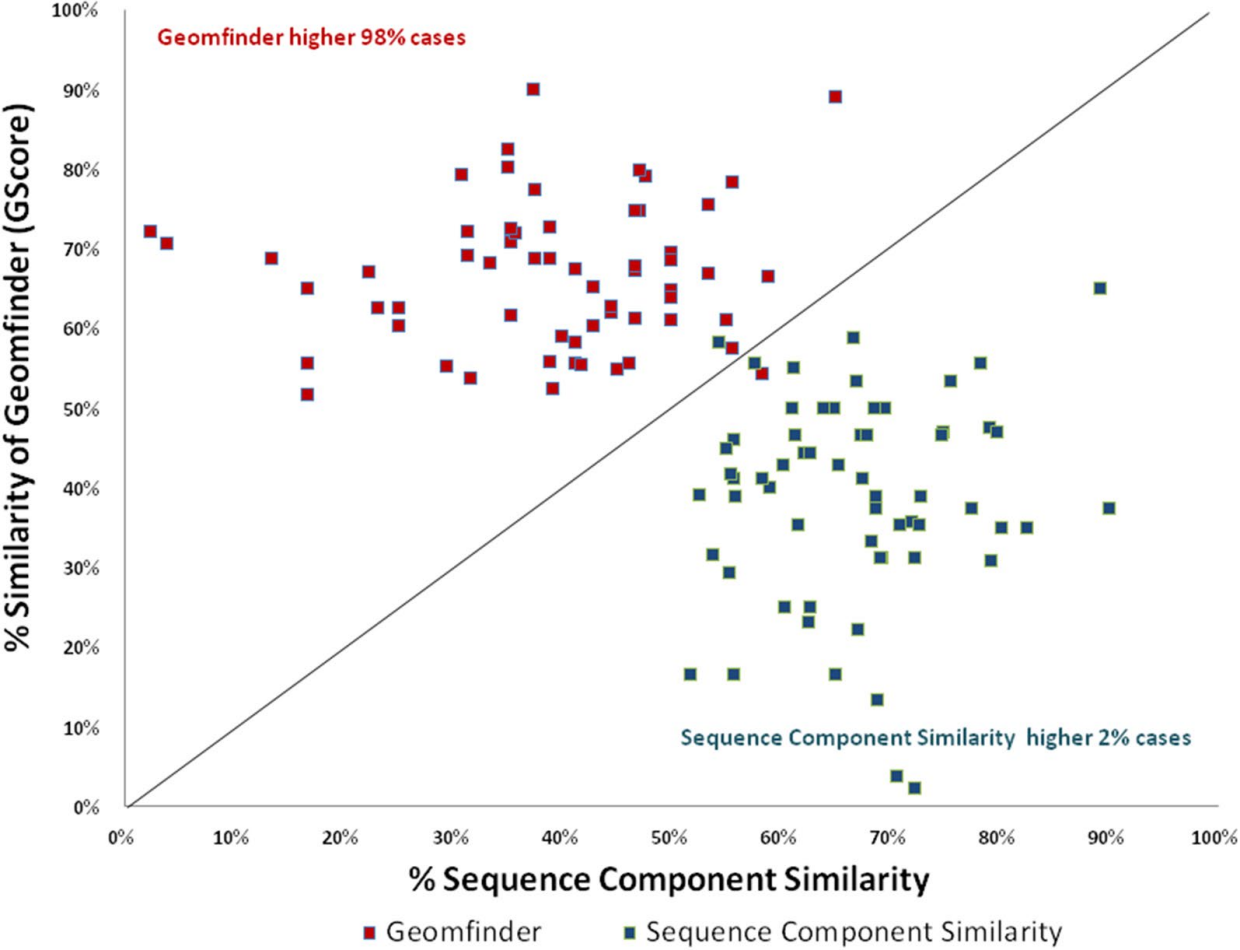
Similarity of the 3D patterns in the Adenosite Triphosphate (ATP) binding proteins. In *red* is represented the higher value of the GScore (Geomfinder) between each pair of 3D patterns related with the ATP binding sites. The Sequence Component Similarity between each pair of ATP target proteins is shown in *blue*. All pair of the evaluated proteins are described in this* scatter plot*

**Fig. 8:**
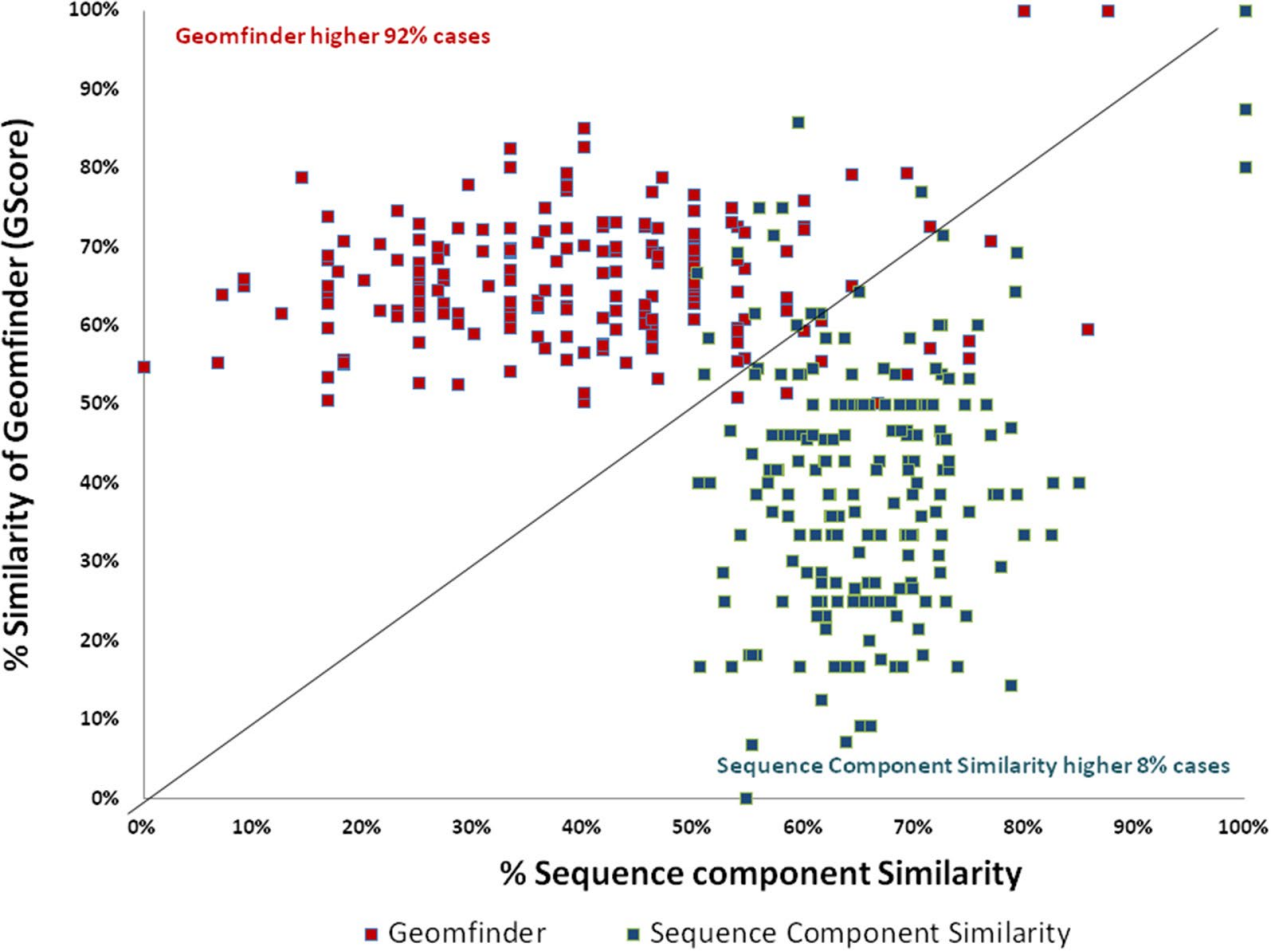
Similarity of the 3D patterns in the Pyridoxal Phosphate (PLP) binding proteins. In *red* is represented the higher value of the GScore (Geomfinder) between each pair of 3D patterns related with the PLP binding sites. The Sequence Component Similarity between each pair of PLP target proteins is shown in *blue*. All pair of the evaluated proteins are described in this scatter plot

**Fig. 9:**
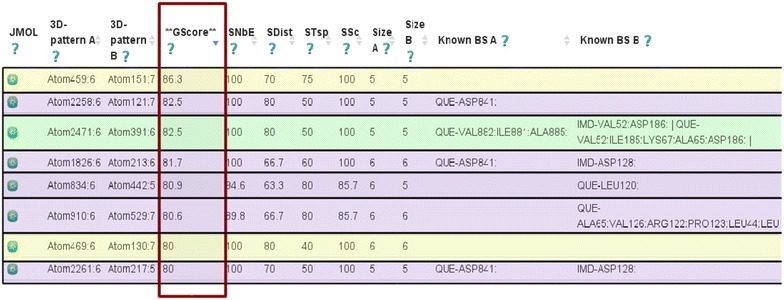
Score values for similar pairs of 3D patterns detected in 1E8W and 2O3P. In this figure, eight pairs of similar 3D patterns are shown. The 3D patterns corresponding to the quercetin binding sites are shown in *green*. The 3D patterns whose residues are located near of the quercetin and imidazole binding sites, are shown in *pink*. The 2 non-obvious similar 3D patterns found, are shown in *yellow*. In the *red box*, are shown the final similarity scores of Geomfinder (GScore)

**Fig. 10:**
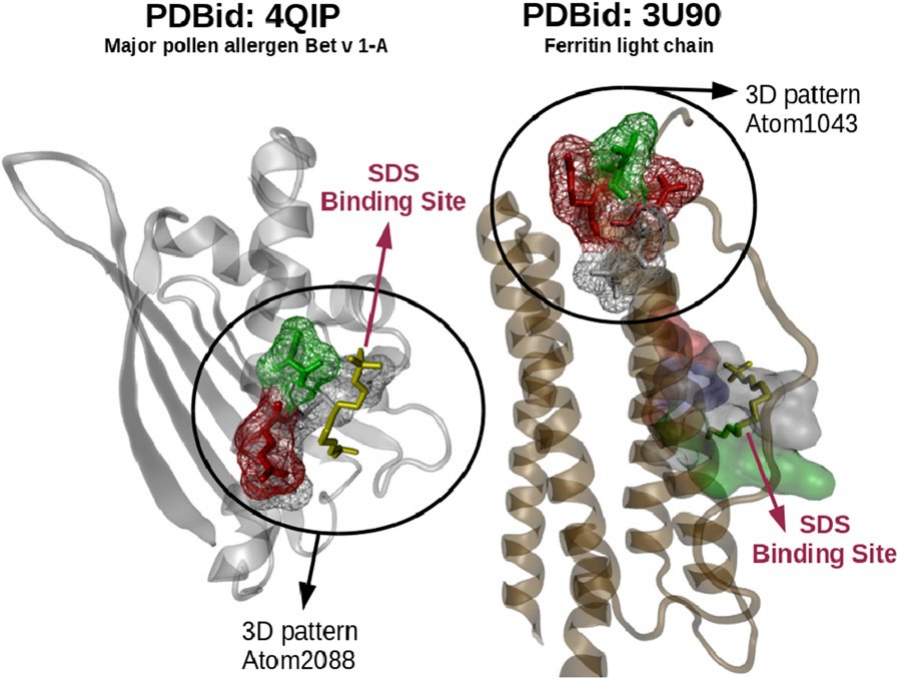
Similar 3D patterns between 4QIP and 3U90. 4QIP and 3U90 proteins are depicted in *gray* and *brown* respectively. SDS is shown in *yellow* and *circles* indicate the most similar pairs of 3D patterns detected

**Fig. 11:**
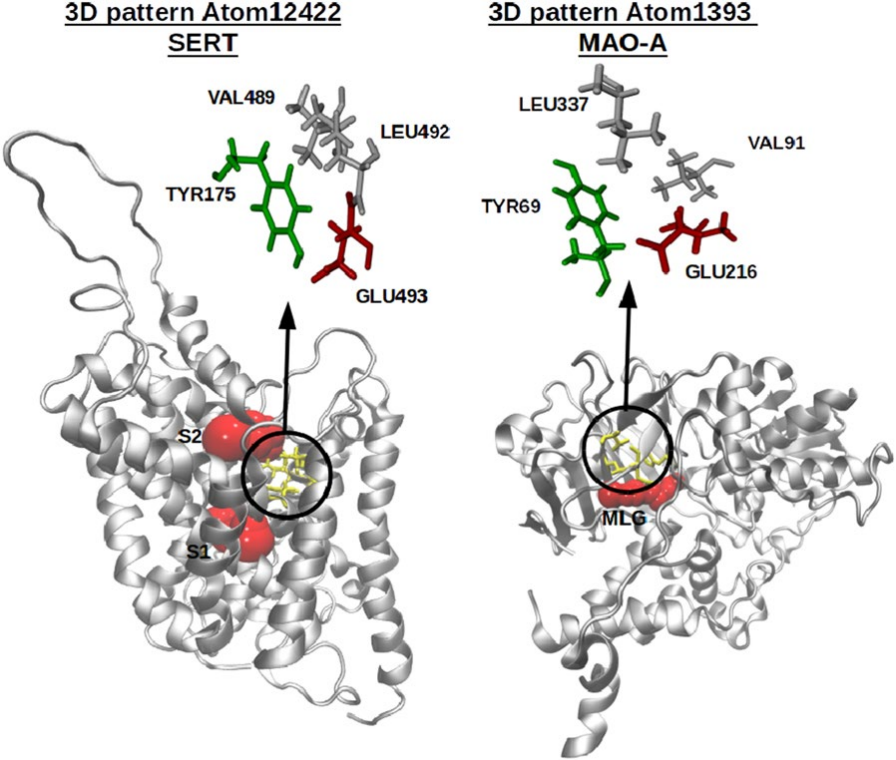
Similar 3D patterns between MAO-A and SERT. The binding sites in SERT (S1 and S2) and MAO-A (MLG) are depicted in *red*. The *circles* represent the identical 3D pattern detected by Geomfinder

### 3D patterns identification

To identify all 3D patterns in each tested protein, the user must define the next parameters:

*Grid Radius* ($G_{r}$): value defined in Å utilized to construct the virtual grid of referential coordinates (Fig. 1). A high value of $G_{r}$, will imply a high amount of referential coordinates in the virtual grid. Thus, the higher the $G_{r}$ value, the more detailed is the exploration of patterns in the proteins (see an example in Table 2).

**Table 1:** Evaluation of the ACR binding site similarities in 1AGM, 3AIC, 1ULV and 3WEM proteins

ACR	GScore	Smith	ProBIS	MultiBind	SiteEngine
Binding Site	Geomfinder (%)	Waterman	Pairwise	Server	Server
Similarity		[48] (%)	[25]	[51]	[52]
1AGM			Alig. Score	33,8856	4279,01
vs	34	32	−3,63	6 same AA	1 same AA
3AIC				of 19	of 13
1ULV				34,2356	4307,17
vs	25	39	Invalid	3 same AA	1 same AA
3WEM			PDB: 3WEM	of 13	of 13

**Table 2:** Virtual Coordinates of Reference. Amount of virtual coordinates of reference in relation with the *Grid Radius* ($G_{r}$) defined by the user to 2O3P and 1E8W proteins

Grid	Virtual	Virtual
Radius	Coordinates	Coordinates
	2O3P	1E8W
1-4	276	859
5	459	1365
6	1197	3531
7	1855	5990
8	2597	8399
9	3421	11,155
*10*	*4535*	*14,702*
11	5906	19,350
12	7353	24,367
13	8897	29,889
14	10,606	35,900
15	12,505	42,838

*Near Threshold* ($N_{t}$): To form a 3D pattern, each residue must be at least $N_{t}$ Å away from a same coordinate of reference in the virtual grid.

*Far Threshold* ($F_{t}$): To form a 3D pattern, each residue must be at most $F_{t}$ Å away from any other.

Hence, $N_{t}$ and $F_{t}$ can be perceived as the dimensional limits of the 3D patterns that are being unveiled. Briefly, if a small value for $N_{t}$ is defined, the detected patterns will include residues that are very close between them. On the contrary, if a higher value for $N_{t}$ is defined, the identified patterns will only include residues that are relatively away from each other. The value of $F_{t}$ represents the maximal distance from the virtual coordinate in which a 3D pattern will be searched, and therefore defines the maximal size of the pattern.

The values of all these parameters, defined by the user, will depend on the aims of the analysis that is being developed. For instance, if the user is interested in searching 3D patterns that could serve as drug binding sites, the $N_{t}$ and $F_{t}$ values should be such that they define a cavity volume that allows to allocate a molecule of a given size. On the other hand, the $G_{r}$ value will determine how detailed the characterization of such pattern will be done (see an example in the Additional file 1: Figure S1).

Once the finding of the 3D patterns is completed, the following four descriptors are calculated as it is shown in the Fig. 2:

- *Distance (Dist):* list of distances between the geometric centers of the side chains of all the residues forming the 3D pattern. Each distance is stored as a number and a pair of letters identifying amino acids (i.e. R5L, where R is Arginine, L is Leucine and 5 is the distance in Å between them). Finally, each 3D pattern will be described by $n(n-1)/2$ terms (where n is the number of residues of the 3D pattern). A similar descriptor has been developed in the algorithm Pocketmatch, where the distances between all residues of each ligand-binding site, measured from three coordinates on the aminoacids, are utilized to calculate the similarity (PMScore) [40].
- *Non-bonded Energy (NbE):* sum of the short and medium range of non-bonded energy of each residue forming the 3D pattern. These physicho-chemical properties were obtained from published data [41]. This type of descriptors have been implemented in the FLAP algorithm [42] in which, through the use of the GRID force field [43], the similarity of binding sites in a pair of proteins, is measured based on the non-bonded energies defined by the Lennard-Jones and Coulomb interactions.
- *Sequence component (Sc):* List of residues forming a 3D pattern. Each residue is tagged into a category defined as: **A: Aliphatic** (*Glycine, Alanine, Valine, Leucine, Isoleucine*), **B: Aromatic** (*Phenylalanine, Tyrosine, Tryptophan*), **C: OH-** (*Serine, Threonine*), **D: Acidic** (*Aspartic Acid, Glutamic Acid*), **E: Acid amide** (*Aspargine, Glutamine*), **F: Basic** (*Arginine, Lysine, Histidine*), **G: Sulphur** (*Cysteine, Methionine*) and **H: Cyclic** (*Proline*). This sequence order-independent approach has been proposed as an efficient form to detect evolutionary relationships [34].
- *Perimeter (Tsp):* list of distances constituting the shortest pathway necessary to go over all the residues lining the 3D pattern. Here the travelling salesman problem algorithm [44] is implemented, and each distance forming the perimeter is stored as a number and a pair of letters identifying amino acids (i.e. R5L, where R is Arginine, L is Leucine and 5 is the distance in Å between them). Finally, each 3D pattern will be described by *n* terms (where n is the number of residues of the 3D pattern). Even though this approach has not been used before to find similar binding sites or patterns, it has been proposed as a competent methodology to clusterize and detect similar folds of protein structures [45].

### Scoring measurements

All pairs of 3D patterns identified in the two tested proteins are compared using an all-versus-all approach. Thus, at the end of the analysis each pair of 3D pattern has a final similarity score (GScore). If this GScore is higher that the threshold defined by the user, the ID of both 3D patterns composing each pair, are linked and stored as a python list element. The GScore is defined as a combination of the similarities (S) of the four descriptors previously mentioned, as stated in the following equation:

1 $$\begin{aligned} GScore = SDist*D_{p} + SNbE*C_{p} + STsp*T_{p} + SSc*S_{p} \end{aligned}$$

In the equation 1, $D_{p}$, $C_{p}$, $T_{p}$ and $S_{p}$ are parameters of Geomfinder that represent the relative contributions of partial similarities of the distance, the non-bonded energy, the perimeter and the sequence component, respectively. These parameters must sum 100 % and are set at 25 % by default (same contribution of each partial similarity to the GScore). If the user is interested in detecting similar 3D patters prioritizing some of these features, for example the non-bonded energies, the relative contribution of each of them can be changed (e.g. $C_{p} = 100$ %, $D_{p} = T_{p} = S_{p} =0$ %; see the Eq. 1).

The terms, *SDist, SNbE, STsp and SSc*, represent the partial similarity associated with each specific descriptor. These similarity values are calculated as the relative changes in each property, according to the following equations:

$$\begin{aligned} SDist &= \frac{\left| {Dist_{A} \cap Dist_{B}}\right| }{ \max (|Dist_{A}|,|Dist_{B}|)}\\ SNbE &= \frac{\min (abs(NbE_{A}),abs(NbE_{B}))}{\max (abs(NbE_{A}),abs(NbE_{B}))}\\ STsp &= \frac{\left| {Tsp_{A} \cap Tsp_{B}}\right| }{\max (|Tsp_{A}|,|Tsp_{B}|)}\\ SSc &= \frac{\left| {Sc_{A} \cap Sc_{B}}\right| }{\max (|Sc_{A}|,|Sc_{B}|)} \end{aligned}$$

where $Dist_{A,B}$, $NbE_{A,B}$, $Tsp_{A,B}$ and $Sc_{A,B}$ are the respective data sets or values previously determined for each pair of 3D patterns compared (sub-indices A and B, represent protein A or B). The “|” symbol is used to denote cardinality.

It should be noted that GScore gives a quantification of similar features that are found between two 3D patterns (e.g. a GScore of 100 % represents identical patterns, whereas 0 % denotes that no similar features exist between the two patterns analyzed). Thus, the GScore is not intended to determine a threshold from which one can establish if two patterns are similar or dissimilar. Instead, it quantifies how similar they are. Therefore the GScore significance will depend on the research question that is being addressed. For instance, in the case of two proteins belonging to the same family and showing very similar global folds, a GScore of 70 % for a given pair of patterns could implicate that such patterns might be exploited for the search of ligands able to discriminate between both proteins. On the other hand, in the case of two completely different proteins, the same GScore value could denote a pair of 3D patterns that might be helpful for the discovery of common ligands.

### System architecture

The architecture of the solution and the essential components are shown in the Fig. 3. This representation is divided into three main items: the presentation layer, the domain layer and the data layer. The presentation layer corresponds to the user’s view, and provides the inputs to the domain layer. It consists of two modules: first, “ParametersView”, which allows the user to enter the necessary data to compute the similarity request (PDB files and general parameters). The second module is the “ResultView”, which gives the results of the comparison to the user. These results are divided into two main views: the similarities scores of each pair of 3D patterns (“TableView”) and the visualization of the protein and/or the 3D pattern structures (“ProteinView”). The domain layer represents the core of Geomfinder and denotes the communication link between the presentation and data layers. The primary components are: GetPDB, PDBProcess, PDBMaker and CompareService (from top to bottom). GetPDB is responsible for getting the PDB files from two possible different sources: PDB files (for homology models) or PDBids (for crystal structures), which are provided by the user. Next, it uploads the files to the server or retrieves the structures from the Protein Data Bank [36]. The PDBprocess module, processes the PDB files from the data layer (previously stored on the server by GetPDB) finding all 3D patterns which are in accordance with the parameters set by the user. As a result, this module generates a list of 3D patterns detected in each PDB file. Additionally, this module interacts with the PDBMaker to generate and save a PDB file in the data layer (this process is carried out for each of the identified 3D patterns). This module has been optimized using python-based multithreading implementation. The Compare-Service receives the lists of 3D patterns generated by the PDBprocess module. With these lists, all similarities scores are calculated, taking into account the parameters provided by the user (ParametersView). In addition, this module generates a file in Json format from the filtered results. Finally, the data layer stores all the files that have been generated in the comparison process.

## Results and discussion

### General evaluation

To evaluate the performance of Geomfinder, a set of 1100 protein structures were obtained from the PDB. Several measurements of partial structure similarity were done with different subsets of proteins. Although in most of the following cases we focused on the analysis of ligand-binding sites, it should be noted that many other pairs of 3D patterns could be studied. In the examples analyzed below, the pairs of proteins assessed were selected arbitrarily and each protein was considered in only one pair (e.g. in a subset of 100 different proteins, 50 pairs of proteins can be defined). All the comparisons done with Geomfinder, for the different pairs of protein structures considered, are shown in the corresponding following figures.

In all evaluations, a filter of GScore value of 50 % was utilized (i.e. only the pairs of 3D patterns with a GScore higher than 50 % were considered in the analyses).

#### Structures of the human enzyme Monoamine Oxidase B

Our first evaluation was done with 38 crystallographic structures of the human Monoamine Oxidase-B (MAO-B; Additional file 2: Table S1). This enzyme is located in the mitochondrial outer membrane and catalyzes the oxidative deamination of biogenic and xenobiotic amines [46]. In all structures available, MAO-B has a co-factor flavin-adenine-dinucleotide (FAD) covalently bound and its location is the reference for a conserved catalytic binding site in this family of proteins [47]. Several compounds which differ in their pharmacodynamics and structure have been co-crystallized with MAO-B (e.g. 1,4-Diphenyl-2-butene, Isatin, n-Propargyl-1(s)-aminoindan, (3R)-3-(prop-2-ynylamino)-2,3-dihydro-1H-inden-5-ol, among others). These differences generate distinct biological responses such as the reversible or the irreversible inhibition of the enzyme. In our tests, Geomfinder was able to detect identical pairs of 3D patterns (pairs of 3D patterns with a $\hbox {GScore} = 100$ %) corresponding to the ligand binding sites of all MAO-B structures (Fig. 4). Since the pairs compared involved the same protein co-crystallized with different inhibitors (Additional file 1: Figure S3), it was not surprising that a high degree of similarity was also found using either global sequence or ligand-independent alignment methods. Thus, a 100 % of similarity was identified with both, the pairwise alignment algorithm of Smith-Waterman implemented on the EMBOSS Website [48] and the CLICK software [49]. Noteworthy, the same performance was not attained using methods, such as PocketMatch [40], which consider the structure of the ligands as the starting point to calculate a similarity score (Fig. 4). Hence, our results confirm the suitability of Geomfinder to recognize, in spite of the presence or absence of ligands, similar 3D patterns (in this case ligand-binding sites) that are effectively similar or identical.

#### Protein structure targets of alpha-acarbose (ACR)

ACR is an anti-diabetic drug used to treat type 2 diabetes mellitus [50]. Its structure corresponds to an oligosaccharide of 5 cyclic units and has been co-crystallized in more than 20 diverse proteins such as glucoamylase II, GacH receptor, glucodextranase, glycoside hydrolase, amylomaltase, among others. Recently ACR has been mentioned as one of the most promiscuous drugs available in the market, and its protein-drug interaction analysis has shown the occurrence of six distinct conformers, which is reflected in 5 clusters of different structural conformations. At the sequence level, more diversity is found and 12 clusters were described [37]. Despite this heterogeneity, Geomfinder was able to detect GScore values higher than 50 % when comparing 3D patterns contained within ACR binding sites in 11 of 13 pairs of proteins evaluated. This finding suggest that the promiscuity of ACR is associated with the existence of similar 3D patterns occurring at the binding sites of the different proteins targeted by the drug. This is in agreement with literature evidence showing that binding site similarity is a crucial feature underlying drug promiscuity [37]. Remarkably, using the same threshold value (50 %), CLICK and PocketMatch software identified structural similarities in 6 and 3 of the 13 pairs compared, respectively (Fig. 5). Furthermore we used the tools ProBIS [25], MultiBind [51] and SiteEngine [52] to evaluate the two pairs of proteins which did not show similar 3D patterns related with the ACR binding site using Geomfinder (PDBid 1AGM versus 3AIC and 1ULV versus 3WEM). As shown in the Table 1, the ACR binding sites of these proteins have different amino acids composition, 3D orientations and physico-chemical properties, confirming the estimations of Geomfinder.

#### Protein structure targets of benzamidine (BEN), adenosine triphosphate (ATP) and pyridoxal phosphate (PLP)

BEN is a reversible competitive inhibitor employed as a ligand to prevent proteases degrading the product of interest in protein crystallography [53], PLP is the most common enzymatic co-factor, being present in a wide number of diverse of proteins and organisms [54] and, as it is well known, ATP plays a fundamental role in a vast amount of chemical reactions in biological systems. Furthermore, these compounds have been co-crystallized with hundreds of proteins such as hydrolases, oxidoreductases, isomerases, ligases, transmembrane proteins, globular proteins, transporters and receptors. In our evaluation, we randomly compared 102 protein targets of BEN, 234 protein targets of ATP and 674 protein targets of PLP (Additional file 2: Table S1). Our results showed that almost in the 70 % of the cases (73 % for BEN, 72.5 % for ATP and 70 % for PLP), Geomfinder found 3D patterns located in the BEN/ATP/PLP binding sites exhibiting *GScore* values higher than 50 %. For comparative purposes, we measured the sequence component similarity of the same pairs of proteins. In this case, to make a fair comparison, only the residues located up to 5 Å from the ligands (i.e. those lining the BEN/ATP/PLP binding sites) were considered. Thus, the sequence component similarity of each pair of proteins was calculated as the percentage of similar residues occurring in both binding sites. Interestingly, these values were in most cases lower than those detected by Geomfinder (Figs. 6, 7, 8). As previously mentioned, the sequence identity in two proteins does not necessarily imply that the spatial organization of the amino acids in each protein site is preserved. Therefore, it is not surprising that Geomfinder (which determines 3D similarities) performed better than a sequence-based method regarding the identification of similar 3D patterns in two given protein structures. Indeed, Geomfinder identified a high degree of similarity between protein 3D patterns showing low sequence identity (Figs. 6, 7, 8), which implies that the residues forming the 3D patterns exhibit Dist, NbA and/or TSP parameter values, that allow to identify them as similar. Thus, this is an important case of evaluation since the similar 3D patterns found by Geomfinder might underlie the binding properties of the ligands analyzed (BEN, ATP, PLP) to structurally unrelated proteins, and also show that the software is able to identify local structural similarities, which cannot be observed at sequence level.

### Detailed evaluation

#### Positive case

We compared the crystal structures of the protein phosphatidyl-inositol 4,5-bisphosphate 3-kinase [PDBid: 1E8W] (961 residues) with the oncogene serine/threonine protein kinase [PDBid: 2O3P] (293 residues). These proteins are very dissimilar and have only 8 % of identity in their primary sequences. The sequence alignment yielded a similarity of 34.3 % with almost 350 residues aligned (including gaps). In our analysis, the initial radius of the distance used to build the virtual grid of coordinates was set in 10 Å. Herewith, 4535 and 14,702 virtual coordinates of reference were created into the protein structures of 2O3P and 1E8W respectively (see an example in the Table 2). This generated more than 66 million of pairs of 3D patterns, which were compared in 5 minutes and 20 seconds. Our results revealed the existence of several pairs of 3D patterns with a GScore higher than 50 %, one of which corresponds to the flavonoid quercetin binding site. This is in agreement with a previous report [55], which showed similar chemical interactions between quercetin and the residues of the binding site in both proteins. Among the highest GScore found, five pairs of these similar patterns were shown to contain residues located near to the co-crystallized ligands quercetin and imidazole, another common ligand. Interestingly, two other non-obvious similar 3D patterns were detected (Fig. 9). In fact, one of these pairs exhibited the highest GScore value (86.3 %) determined after comparison of both proteins. The patterns in this pair were detected from the virtual reference coordinate Atom459 (in 1E8W) and Atom151 (in 2O3P), and showed values of 100 % of similarity for the non-bonded energies (SNbE) and the sequence component (SSc) parameters. It should be noted that in order to align the sequences of these 3D patterns (ASP637, PRO671, SER636, GLU638 and ARG641 to the Atom459, and ASP114, PRO113, SER115, GLU70 and ARG112 to Atom151), a sequence-based method must incorporate at least 40 gaps, with the corresponding decrease of the alignment significance.

#### Negative case

We compared the structures of the intracellular apoferritin protein from *Equus caballus* [PDBid: 3U90] (174 residues) with the major birch pollen allergen Bet v1 protein from *Betula pendula* [PDBid: 4QIP] (159 residues). Both proteins have been co-crystallized with sodium dodecyl sulfate (SDS) and share a 35 % of their amino acids sequences. In our tests, Geomfinder did not find similar 3D patterns corresponding to their SDS binding sites. This result indicates that even though both proteins share a common ligand, the binding sites and the binding mode of SDS at these sites are not similar. Nevertheless, Geomfinder identified some similar 3D patterns in both proteins. Thus, the best GScore (67,7 %) detected patterns defined from the virtual reference Atom1043 (3U90) and Atom2088 (4QIP). Interestingly, the 3D pattern denoted by the virtual reference Atom2088 (4QIP) was located in the SDS binding site whereas the 3D pattern defined by the virtual reference Atom1043 (3U90) was located in an extracellular zone of the protein (Fig. 10). After an evaluation with the software MetaPocket [56], a possible ligand-binding site was identified in the same zone that was defined by the virtual reference Atom1043 in the protein 3U90 (residues ALA14 and ALA15; Additional file 1: Figure S2). This result suggests that these proteins might still share a similar binding site, and could interact with some currently unknown common ligands.

#### Uncommon case

In an additional evaluation, we analyzed the crystal structure of the human monoamine oxidase A (MAO-A) [PDBid: 2BXS] co-crystallized with the selective and irreversible inhibitor clorgiline (MLG), and a homology model of the human serotonin transporter (SERT), built using the structure of the Drosophila melanogaster dopamine transporter (DAT) [PDBid: 4M48], as template. It has been shown that two putative ligand binding sites exist in SERT, named S1 and S2 [57–59], whereas a single substrate binding site is found in MAO-A [47]. Both proteins are considerably different from a structural point of view, and while SERT is a transmembrane protein belonging to SLC6 family, MAO-A is an outer mitochondrial membrane bound flavoprotein, with the FAD cofactor covalently bound to the enzyme. Their global sequence identity is only of 3.9 % while the local sequence similarity shows a 34 % in a segment of 55 aligned residues including 19 gaps. Nevertheless, the neurotransmitter serotonin (5-HT) is a common ligand and the physiological actions of both proteins are related with the regulation of adequate levels of 5-HT in the synaptic cleft. In spite of the low sequence similarity, Geomfinder was able to detect several similar 3D patterns between SERT y MAO-A, one of which correspond to the MLG binding site in MAO-A and the binding site S2 of SERT. These 3D patterns, defined by the virtual reference Atom1393 (in MAO-A) and Atom12422 (in SERT), have a GScore value of 100 % (Fig. 11). We designated this case as “uncommon” since it shows that Geomfinder is able to find identical 3D patterns in binding sites belonging to proteins with highly different sequences, structures, genetic origin, tissue distribution and catalytic activities. In this particular case, SERT and MAO-A similarities found suggest the existence of some degree of structural convergence between both proteins, which could be related with the recognition of the common substrate.

## Conclusions

Geomfinder is a an intuitive, flexible and alignment-free web server to detect all similar 3D patterns between any pairs of protein structures, which can come from both, X-ray experiments or homology models. The similarity score of Geomfinder (GScore) is calculated as the sum of the relative contribution of the partial similarities of different features of the 3D patterns, such as distance, non-bonded energy, sequence-component similarity and the perimeter. The latter had not been used thus far by the current available structured-based methods that measure local structural similarities, and represents an efficient inclusion of an algorithm commonly used in areas such as transport, business and logistics applications, into a biochemical context. Several examples were analyzed and millions of measurements in almost 1100 protein structures were done. Our results confirm the sensitivity of Geomfinder to detect all similar 3D pattern related to the binding sites of MAO-B, even in those cases where the structure of ligands were highly different. The assessment of protein targets of ACR, PLP, BEN and ATP, revealed the relevance of finding partial similarities at structural level, which is unaffected by the natural divergence of the amino acids sequences. In addition, the detailed evaluation of three specific cases was described. These analyses showed the versatility of Geomfinder which was able to discriminate between similar and different 3D patterns related to binding sites of common substrates in a range of diverse proteins. Remarkably, Geomfinder detected identical 3D patterns associated to the binding sites of a common substrate in two fylogenetically distant proteins such as SERT and MAO-A. In this context, our software can be useful for determining potential druggable sites in unrelated proteins, which is a primary input for the structure-based rational design of drugs with a polypharmacological profile. Interestingly, as our approach is ligand-independent, the similar 3D patterns identified by Geomfinder could represent unanticipated ligand binding sites that might be associated to very different functions in each protein. This gives an unusual opportunity for exploring the chemical space in the search of molecules that could fit and interact at these cavities. For instance, one can envision novel pharmacological properties for a compound simultaneously affecting the activity of two proteins if it binds to an allosteric site in one target and disrupts protein interactions in the other one. In addition, based on the occurrence of 3D pattern similarities and the possible existence of similarities between more than two structural motifs, the results of Geomfinder help to unveil more subtle connections between proteins, and therefore be useful for novel procedures of protein classification. Thus, for instance, a group of proteins classified as functionally related on the basis of a similar catalytic activity, might be further sub clustered if the similarities between other 3D patterns are considered.

Even though in most of the cases analyzed, Geomfinder exhibited a better/similar performance when compared to other tools, it should be stressed that an exhaustive benchmarking was not intended, since our software executes a type of analysis that differs from those carried out by other available programs. Thus, the ability of Geomfinder to detect a higher number of pairs of proteins with similar 3D patterns associated to the binding of a common ligand (e.g. the case of ACR), seems to be more related to a conceptual difference rather than a better technical performance. In this context, the identification and comparison of 3D patterns, which are usually smaller than the whole cavitiy defining a binding site, can reveal similarities that are not detected by current ligand-dependent or independent algorithms which align the most similar three-dimensional substructure between a pair of protein structures [25, 49].

 Finally, it should be noted that Geomfinder is not intended for simultaneous multiple comparisons in a large number or families of proteins. However, it can be particularly useful in cases such as the structure-based design of multitarget drugs, where a detailed analysis of 3D patterns similarities between a few selected protein targets is essential.

## Availability and requirements

- Project name: Geomfinder
- Project home page: Geomfinder can be executed in http://appsbio.utalca.cl/geomfinder/. The source files of the entire Proyect is freely and anonymously available in the following Bitbucket repository: https://bitbucket.org/gnunezv/geomfinder
- Operating system(s): Platform independent
- Programming language: Python, JavaScript, PHP, HTML.
- Other requirements: The web-browser must have the Java Plugin activated.
- License: GNU GPL.
- Any restrictions to use by non-academics: Formal authorization by the authors is needed for commercial use of Geomfinder.

## Additional files

10.1186/s13321-016-0131-9 Supplementary data.
10.1186/s13321-016-0131-9 Table of the PDBid of the analyzed proteins.

## Abbreviations

3D: three-dimensional
PDB: protein data bank database
Gr: grid radius parameter
Nt: near threshold parameter
Ft: far threshold parameter
Dist: descriptor of the distances
NbE: descriptor of the non-bonded energies
Sc: descriptor of the component of the sequences
Tsp: descriptor of the pathway of the perimeter
GScore: final similarity score of Geomfinder
Dp: relative contribution of the distances in the final similarity score
Cp: relative contribution of the non-bonded energies in the final similarity score
Tp: relative contribution of the perimeter in the final similarity score
Sp: relative contribution of the sequence component in the final similarity score
SDist: descriptor of the similarity of the distances
SNbE: descriptor of the similarity of the non-bonded energies
SSc: descriptor of the similarity of the component of the sequences
STsp: descriptor of the similarity of the the pathway of the perimeter
FAD: flavin-adenine-dinucleotide
MAO-B: monoamine oxidase B
ACR: alpha-acarbose
BEN: benzamidine
ATP: adenosine triphosphate
PLP: pyridoxal phosphate
SDS: sodium dodecyl sulfate
MAO-A: monoamine oxidase A
MLG: clorgiline
SERT: serotonin transporter
DAT: dopamine transporter
SLC6: solute carrier family 6
5-HT: serotonin
FONDECYT: Fondo Nacional de Desarrollo Científico y Tecnológico
