# Supplementary material for: Geomfinder: a multi-feature identifier of similar three-dimensional protein patterns: a ligand-independent approach
Source: J Cheminform. 2016 Apr 18;8:19. doi: 10.1186/s13321-016-0131-9 (PMC4834829; doi:10.1186/s13321-016-0131-9)
Supplement: Supplementary file 1 — 10.1186/s13321-016-0131-9 Supplementary data. [file 13321_2016_131_MOESM1_ESM.pdf]

Figure S1: Schematic representation of the parameters Gr (Grid Radius), Nt (Near Threshold) and Ft (Far Threshold).

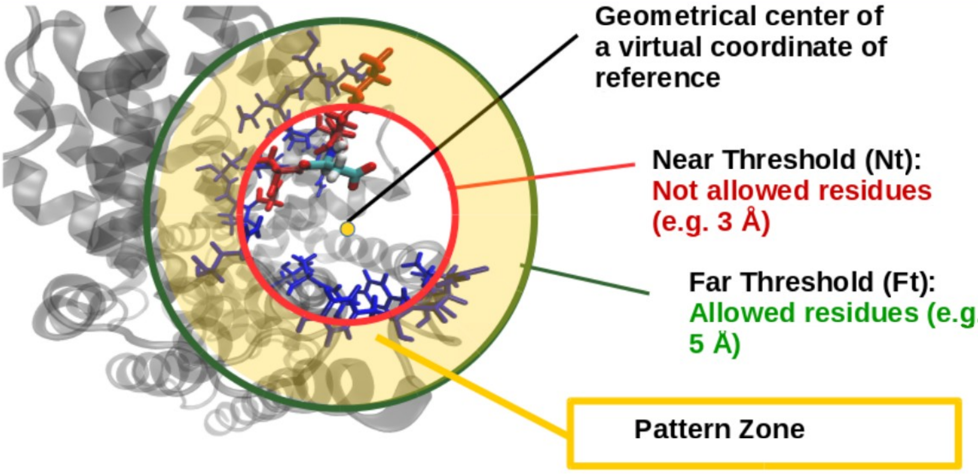

<u>Examples</u>

Near Threshold: 3Å, Far Threshold: 6Å

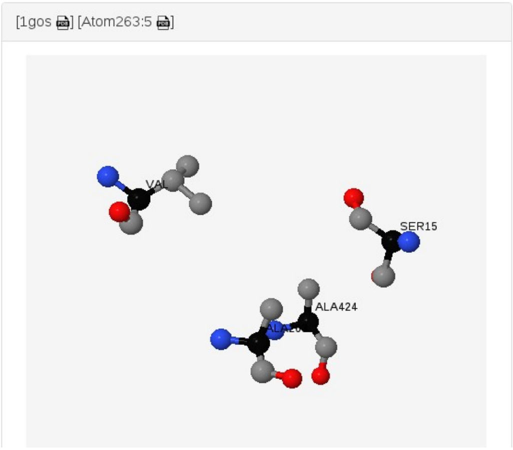

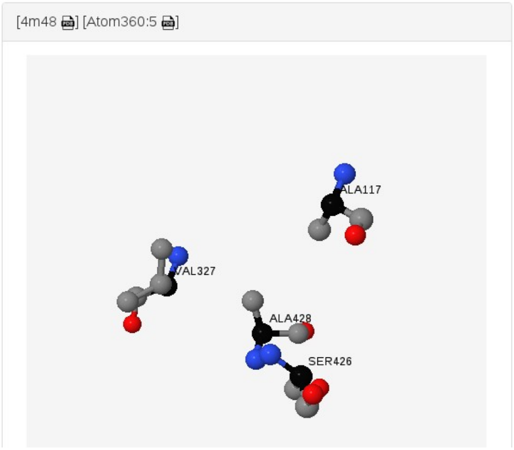

Near Threshold: 5Å, Far Threshold: 15 Å

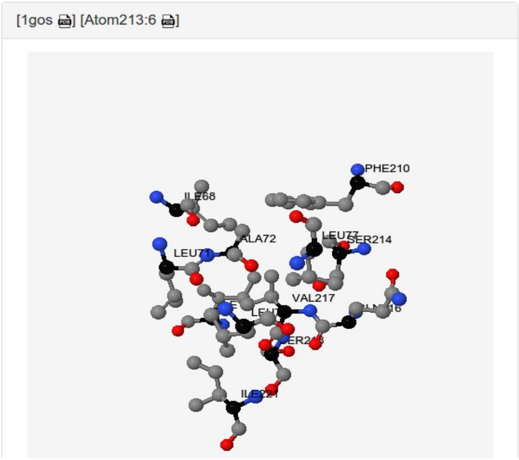

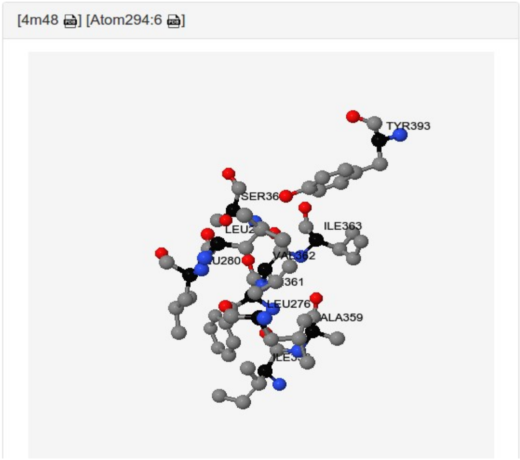

Figure S1: This figure shows a schematic representation of how the results can be modulated by the choice of different values for the parameters Gr (Grid Radius), Nt (Near Threshold) and Ft (Far Threshold).

Figure S2: Potentials ligand-binding sites in 3U90.

```
The potential 3 ligand binding sites in your protein:
--------------------------------------------------------------------------------
HEADER binding site ID: 1
RESI      THR_A^29^     LEU_A^33^     TRP_A^89^     ALA_A^98^     ALA_A^99^
RESI      LEU_A^102^    LYS_A^97^     VAL_A^101^    SER_A^85^     GLN_A^86^
RESI      SER_A^105^    PRO_A^84^     ARG_A^25^     ALA_A^26^     LYS_A^83^
RESI      LEU_A^22^     LEU_A^106^    TYR_A^28^     GLN_A^82^     LEU_A^24^
RESI      LEU_A^81^     ASN_A^21^     ASP_A^94^
HEADER binding site ID: 2
RESI      PHE_A^50^     LYS_A^143^    HIS_A^147^    LYS_A^139^    LEU_A^140^
RESI      HIS_A^49^     GLU_A^53^     LEU_A^54^     GLU_A^136^    LYS_A^142^
RESI      VAL_A^138^    ARG_A^52^     GLU_A^57^     LYS_A^58^     GLU_A^60^
RESI      GLY_A^61^     ARG_A^64^     HIS_A^132^    PHE_A^133^    TYR_A^23^
RESI      GLU_A^63^     LEU_A^171^    GLY_A^46^     THR_A^170^
HEADER binding site ID: 3
RESI      ASN_A^17^     VAL_A^20^     LEU_A^66^     ALA_A^76^     LEU_A^77^
RESI      PHE_A^78^     ASN_A^21^     VAL_A^16^     GLN_A^69^     LEU_A^24^
RESI      GLN_A^79^     ARG_A^18^     LEU_A^81^     ALA_A^14^     ASP_A^80^
RESI      LEU_A^22^     LEU_A^19^     ARG_A^25^     ALA_A^15^     ALA_A^109^
RESI      ASP_A^112^
```

Figure S2: In this figure, three potential ligand-binding sites are shown. These sites were predicted in the protein 3U90 by the Metapocket Software. In the red square is tagged the site that corresponds to the similar 3D pattern detected by Geomfinder.

Figure S3: Structure of Monoamine Oxidase B Co-cristalized compounds.

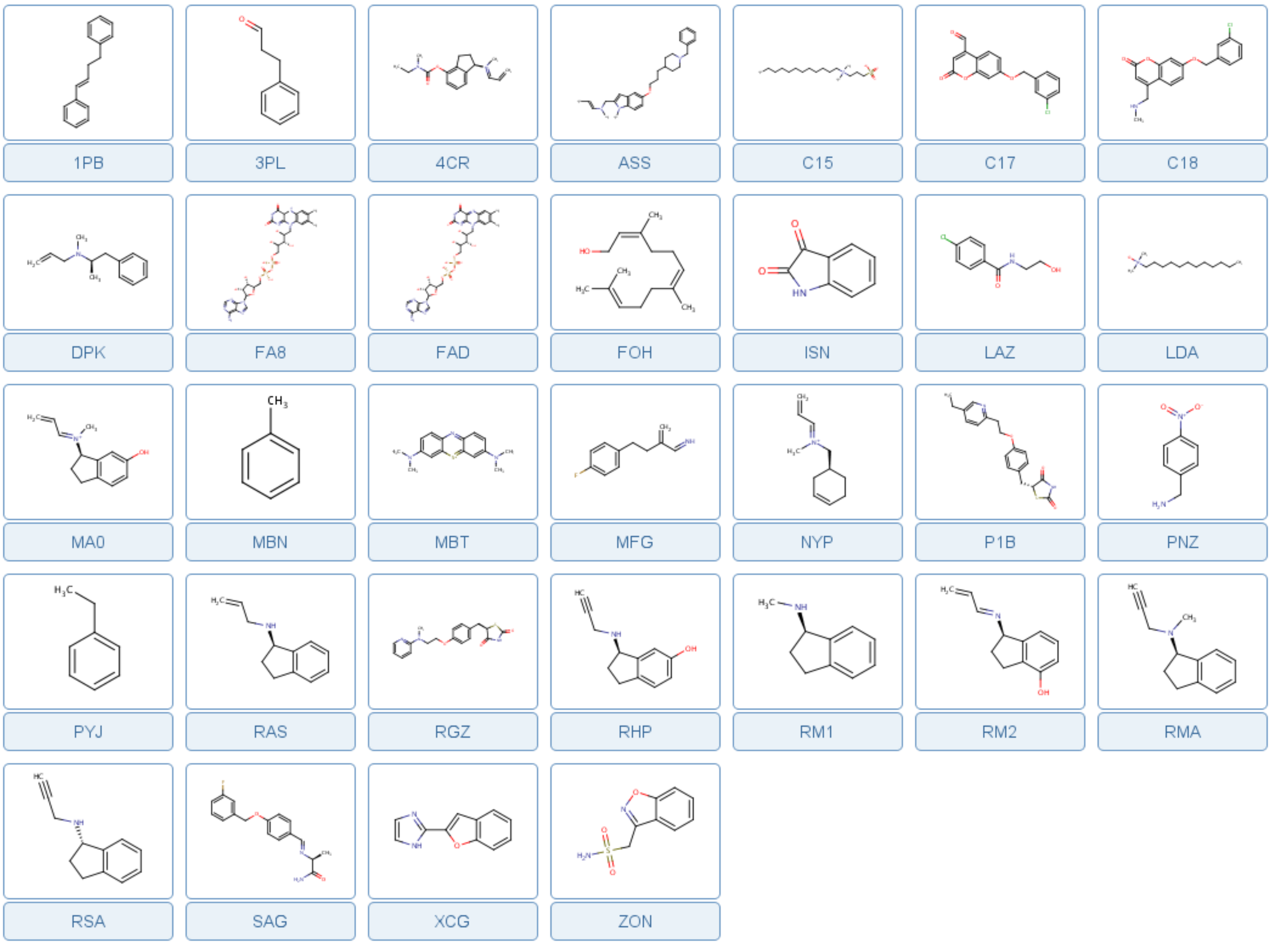

Figure S3: In this figure, 32 different compounds which have been co-crystallized with the Monoamine Oxidase B enzyme. The three letter code, which is shown in the blue squares below of each structure, correspond to the ID of the ligands stored in the Protein Data Bank database.
