# Supplementary material for: Geomfinder: a multi-feature identifier of similar three-dimensional protein patterns: a ligand-independent approach
Source: J Cheminform. 2016 Apr 18;8:19. doi: 10.1186/s13321-016-0131-9 (PMC4834829; doi:10.1186/s13321-016-0131-9)
Supplement: Supplementary file 2 — 10.1186/s13321-016-0131-9 Table of the PDBid of the analyzed proteins. [file 13321_2016_131_MOESM2_ESM.pdf]

Table S1: Data set of the pairs of evaluated proteins

Ligand	PDBid of the pair of proteins evaluated	Amount Of PDBs	Pairs Tested
Acarbose (ACR)	1esw/3bc9 ; 1gah/3jyr ; 1k1y/3jzj ; 1kxh/3k8m ; 1lf9/3top ; 1mxd/3w37 ; 1mxg/3wel ; 2f6d/3wen ; 2owc/3weo ; 2qmj/4uac	26	13
Benzamidine (BEN)	1bit/3b3j ; 1bty/3bg8 ; 1cc7/3gy7 ; 1cc8/3i78 ; 1ce5/3iti ; 1dpo/3m7o ; 1eax/3mfj ; 1h4w/3mi4 ; 1j14/3nq8 ; 1j15/3nqv ; 1j16/3p8g ; 1j8a/3plb ; 1l2e/3ptb ; 1lo6/3pwb ; 1lpu/3qk1 ; 1lr4/3rxe ; 1mbq/3rxq ; 1nsa/3rxu ; 1oss/3rxv ; 1s0r/3t25 ; 1wri/3t26 ; 1zhm/3t27 ; 1zhp/3t28 ; 1zhr/3t29 ; 2aiq/3tay ; 2ayw/3tpk ; 2blv/3unq ; 2blw/3unr ; 2bmv/3uy9 ; 2ckr/3zsn ; 2cks/4dso ; 2eek/4e2k ; 2j9n/4edg ; 2o8u/4edk ; 2oq5/4edr ; 2oxs/4edt ; 2tbs/4edv ; 2tio/4ee1 ; 2trm/4emn ; 2y46/4eqm ; 2y5z/4i8g ; 2y8d/4i8h ; 2zpq/4i8j ; 2zpr/4i8k ; 2zps/4i8l ; 3atl/4ixu ; 3b3j/4jpu ; 3bg8/4n8z ; 3gy7/4ncy ; 3i78/4nfe ; 3iti/4nvc	102	51
Adenosine triphosphate (ATP)	1a0i/3tuv ; 1ayl/3tw3 ; 1b38/3v01 ; 1b39/3v04 ; 1d9z/3vez ; 1dv2/3vnq ; 1dy3/3vvh ; 1e8x/3w1g ; 1fmw/3w5o ; 1gol/3whk ; 1hck/3wqu ; 1hi1/3wt0 ; 1hp1/3wv9 ; 1ii0/3zcn ; 1ijj/4a06 ; 1j09/4a07 ; 1j7k/4a2a ; 1ji0/4a6x ; 1jjv/4an3 ; 1jkn/4aw0 ; 1kax/4aw1 ; 1kay/4azw ; 1kaz/4b0s ; 1mb9/4b9q ; 1mo8/4beb ; 1n75/4bec ; 1nge/4ct1 ; 1ngf/4ct2 ; 1ngg/4dth ; 1ngh/4dtl ; 1obd/4dx2 ; 1ol6/4dxl ; 1os1/4ed4 ; 1phk/4edg ; 1q97/4ej7 ; 1qhg/4ffl ; 1qz5/4ffm ; 1qz6/4ffn ; 1r0x/4ffo ; 1r0z/4ffr ; 1r10/4fg7 ; 1s22/4fg8 ; 1tqp/4fg9 ; 1u5r/4fhx ; 1ua2/4fi1 ; 1uev/4fo0 ; 1vc9/4fut ; 1vjc/4fvq ; 1vjd/4fvr ; 1wua/4gni ; 1xdn/4gt3 ; 1xf9/4gxq ; 1xfa/4gxr ; 1xmj/4hdh ; 1xsc/4ife ; 1yfr/4itm ; 1ytm/4k41 ; 1yun/4lya ; 1zao/4mwh ; 1zp9/4n1a ; 2aru/4nh0 ; 2asm/4o4d ; 2aso/4o4e ; 2asp/4o4f ; 2b6f/4oab ; 2bbo/4ohw ; 2bbs/4pl7 ; 2bbt/4pla ; 2biy/4qdi ; 2bup/4qf5 ; 2c8v/4qnr ; 2c96/4rh7 ; 2c9c/4rqk ; 2cbz/4rqv ; 2faq/4rrv ; 2fgj/4rv7 ; 2fgk/4ttq ; 2fxu/4u0m ; 2gwj/4wae ; 2hf4/4wb6 ; 2hix/4wc0 ; 2hs0/4wh3 ; 2ijm/4wzy ; 2ily/3g59 ; 2ivp/3h1q ; 2ixg/3hav ; 2iyw/3hbt ; 2jk8/3hmn ; 2pzf/3mmv ; 2pzg/3mn6 ; 2q0d/3mn9 ; 2q0r/3nh9 ; 2q0u/3o8l ; 2qb8/3os3 ; 2qk4/3pgk ; 2r9v/3pp1 ; 2rsf/3q53 ; 2vcp/3q60 ; 2vyp/3qb0 ; 2w00/3qx9 ; 2w02/3r1r ; 2w5g/3si7 ; 2w5i/3sl2 ; 2x15/3t0z ; 2ych/3t54 ; 2yw2/3t8o ; 2zan/3tut ; 3a7h/3dnt ; 3a8t/3dy7 ; 3a8w/3e8n ; 3ar4/3efs ; 3att/3ehg ; 3bu5/3eks ; 3c5e/3eku ; 3cyi/3el2 ; 3dgl/3eps ; 3dkc/3fcc	234	117

Pyridoxal Phoshate (PLP)	1a3g/2d61 ; 1aam/2d63 ; 1aaw/2d64 ; 1ahe/2d65 ; 1ahf/2d66 ; 1ahp/2d7y ; 1ahx/2d7z ; 1ahy/2dab ; 1aka/2dgk ; 1ari/2dgl ; 1ars/2dgm ; 1art/2dh5 ; 1asa/2dkb ; 1asb/2dkj ; 1asf/2dr1 ; 1asg/2dy3 ; 1asn/2e7i ; 1axr/2e7j ; 1ay4/2eco ; 1ay5/2ecp ; 1ay8/2ecq ; 1b4d/2efy ; 1b4x/2egy ; 1b54/2eh6 ; 1b5o/2eiy ; 1b5p/2ej3 ; 1b8g/2eo5 ; 1b9h/2f3p ; 1bj4/2f3q ; 1bjo/2f3s ; 1bt4/2f3u ; 1bx3/2fet ; 1c0n/2ff5 ; 1c4k/2ffr ; 1c50/2fni ; 1c7g/2fyf ; 1c7n/2gpa ; 1c8k/2gpb ; 1c8l/2hdk ; 1cj0/2hg8 ; 1ct5/2hgw ; 1czc/2hgx ; 1cze/2hhf ; 1d2f/2hzp ; 1d6s/2isq ; 1daa/2j66 ; 1dgd/2jc3 ; 1dge/2jis ; 1dje/2jje ; 1dju/2jjg ; 1dka/2jjh ; 1dty/2nmp ; 1e1y/2nv9 ; 1e4o/2o1b ; 1e5f/2oo0 ; 1ecx/2ord ; 1eg5/2p69 ; 1ekf/2pb0 ; 1ekp/2pb2 ; 1ekv/2pqm ; 1elq/2pri ; 1em6/2prj ; 1eqb/2qgh ; 1et0/2qll ; 1exv/2qn7 ; 1f2d/2qn8 ; 1f3t/2qn9 ; 1fa9/2rjg ; 1fc0/2rkb ; 1fc4/2sfp ; 1fcj/2skc ; 1fg3/2skd ; 1fs4/2ske ; 1ftq/2tod ; 1ftw/2uzp ; 1fty/2vd8 ; 1fu4/2vgs ; 1fu7/2vgt ; 1fu8/2vgu ; 1g2w/2vgv ; 1g4v/2vgw ; 1g4x/2vi8 ; 1g76/2vi9 ; 1g77/2via ; 1g78/2vib ; 1g79/2vmn ; 1g7w/2vmo ; 1g7x/2vmp ; 1gbn/2vmq ; 1gc3/2vmr ; 1gc4/2vms ; 1gck/2vmt ; 1gd9/2vmu ; 1gde/2vmv ; 1gew/2vmw ; 1gex/2vmx ; 1gfz/2vmy ; 1gg8/2vmz ; 1ggn/2w7d ; 1gpa/2w7e ; 1gpb/2w7f ; 1gpy/2w7g ; 1h0c/2w7h ; 1h1c/2w7i ; 1h5u/2w7j ; 1hkv/2w7k ; 1hlf/2w7l ; 1i1k/2w7m ; 1i1l/2wk8 ; 1i1m/2wk9 ; 1i29/2x5d ; 1i2k/2x5f ; 1i43/2xh1 ; 1i48/2y4r ; 1iax/2yct ; 1iay/2yhk ; 1ibj/2yku ; 1iji/2ykx ; 1ix6/2yky ; 1ix7/2yob ; 1ix8/2yrr ; 1iyd/2yxx ; 1j0a/2z1y ; 1j0c/2z1z ; 1j0e/2z20 ; 1j32/2z67 ; 1jbq/2zb2 ; 1jf9/2zgi ; 1jnw/2zp7 ; 1js3/2zsj ; 1js6/2zuk ; 1k06/2zy2 ; 1k08/2zy3 ; 1kkj/2zy4 ; 1kkp/2zy5 ; 1kl1/3a2b ; 1kl2/3a9x ; 1kl7/3a9y ; 1kmj/3a9z ; 1kmk/3aat ; 1knw/3amv ; 1ko0/3anu ; 1kti/3anv ; 1l5q/3aov ; 1l5r/3aow ; 1l5s/3ath ; 1l5v/3awn ; 1l5w/3awo ; 1l6i/3b1c ; 1l7x/3b1d ; 1lk9/3b8t ; 1lkc/3b8u ; 1ls3/3b8v ; 1lwn/3b8w ; 1lwo/3bb8 ; 1m32/3bd7 ; 1m4n/3bd8 ; 1m54/3bda ; 1mdx/3bm5 ; 1mdz/3bv0 ; 1mgv/3c5q ; 1mly/3ceh ; 1mlz/3cej ; 1n2t/3cem ; 1n31/3co8 ; 1n8p/3cog ; 1noi/3csw ; 1noj/3dd1 ; 1nok/3dds ; 1nrg/3ddw ; 1o4s/3dod ; 1o61/3dtg ; 1oas/3du4 ; 1oat/3dwg ; 1ohv/3dwi ; 1ohw/3dxv ; 1ohy/3dxw ; 1ord/3dyd ; 1p29/3e5p ; 1p2d/3e9k ; 1p2g/3ei8 ; 1p3w/3eib ; 1p4g/3ele ; 1p4h/3f9t ; 1p4j/3fcr ; 1p5j/3fdd ; 1pg8/3g8m ; 1pmm/3gju ; 1qgn/3gpb ; 1qir/3hmk ; 1qis/3hqt ; 1qit/3hy8 ; 1qj3/3i5t ; 1qj5/3if2 ; 1qm5/3ihj ; 1qu4/3ii0 ; 1qz9/3isl ; 1rcq/3jz6 ; 1rfu/3k28 ; 1rqx/3k7y ; 1rv3/3keu ; 1rv4/3kgw ; 1rvu/3kki ; 1s07/3kow ; 1sf2/3kp1 ; 1sft/3l6b ; 1szs/3l6c ; 1szu/3l8a ; 1t3i/3lv2 ; 1tar/3lvj ; 1tat/3lvk ; 1tdj/3lvm ; 1twi/3ly1 ; 1tyz/3meb ; 1tz2/3n29 ; 1tzj/3n2o ; 1tzk/3nys ; 1tzm/3nzp ; 1u08/3o05 ; 1uim/3pc2 ; 1uin/3ppl ; 1uzu/3qbo ; 1v2d/3rbf ; 1v2e/3tat ; 1v2f/3tft ; 1v71/3tqx ; 1v72/3uyy ; 1v8z/3uzb ; 1ve1/3uzo ; 1ve5/3vax ; 1vef/3vbe ; 1vfh/3vom ; 1vjo/3vsa ; 1vp4/3vsc ; 1w23/3vsd ; 1w3u/3wqc ; 1w8g/3wqd ; 1wkv/3wqe ; 1wrv/3wqf ; 1wst/3wqg ; 1wtc/3zcp ; 1wut/3zcq ; 1wuy/3zcr ; 1wv0/3zcs ; 1wv1/3zct ; 1ww2/3zcu ; 1ww3/3zcv ; 1xc7/3zei ; 1xey/3zrp ; 1xfc/3zzj ; 1xi9/4a0f ; 1xkx/4a0g ; 1xl0/4a0h ; 1xl1/4a0r ; 1xoi/4a3q ; 1xql/4a6t ; 1yaa/4adb ; 1ygp/4adc ; 1yjs/4add ; 1yjy/4aec ; 1yjz/4ah3 ; 1yoo/4ao9 ; 1z3z/4atp ; 1z62/4atq ; 1z7w/4azj ; 1zob/4azk ; 1zod/4b98 ; 2a1h/4b9b ; 2a5h/4beq ; 2abj/4beu ; 2amv/4bf5 ; 2aq6/4bmk ; 2asv/4bq0 ; 2ati/4bqe ; 2av6/4bqf ; 2aw3/4bqi ; 2ay1/4cbr ; 2ay2/4cbs ; 2ay3/4ce5 ; 2ay4/4chi ; 2ay5/4cmd ; 2ay6/4coo ; 2ay7/4ctm ; 2ay8/4ctn ; 2ay9/4cto ; 2azd/4cvq ; 2bhs/4cxq ; 2bhx/4cxr ; 2bi1/4daa ; 2bi2/4dgt ; 2bi3/4dq6 ; 2bi5/4e1o ; 2bi9/4eb5 ; 2bia/4eb7 ; 2bie/4emy ; 2big/4f4f ; 2byj/4fl0 ; 2byl/4gpb ; 2c0r/4gsa ; 2c4m/4h6d ; 2c7t/4je5 ; 2can/4jey ; 2cft/4jf1 ; 2ch1/4k6n ; 2ch2/4l0d ; 2cin/4l0o ; 2cjd/4l27 ; 2cjh/4l28 ; 2cog/4l3v ; 2coi/4lma ; 2coj/4lmb ; 2cst/4lw2 ; 2ctz/4lw4 ; 2d1f/4m2j ; 2d5y/4m2k ; 4w1x/4m2m ; 4w5k/4n0w ; 4wbt/4nog ; 4y0h/4o6z ; 4yua/4obu ; 4z5x/4ot8 ; 4zlv/4otl ; 4zwm/4pb3 ; 5bj3/4pb4 ; 5bj4/4pb5 ; 5daa/4pcu ; 5eaa/4pff ; 5gpb/4pfn ; 6gpb/4ppm ; 7aat/4qgr ; 7gpb/4qys ; 7odc/4rkd ; 8aat/4s1i ; 8gpb/4uox ; 9gpb/4uoy ; 4w1v/4uqv ; 4w1w/4v15	674	337
MAO-B Proteins	1gos-NYP/1ojd-LDA ; 1oj9-1PB/1s2q-RAS ; 1oja-ISN/1s2y-RSA ; 1ojc-LAZ/1s3b-RMA ; 2bk3-FOH/1s3e-RHP ; 2bk4-RAS/2c67-RM1 ; 2bk5-ISN/2c70-PNZ ; 2byb-DPK/2c72-RSA ; 2c64-MA0/2c73-RSA ; 2c65-4CR/2c75-RSA ; 2c66-RM2/2c76-RSA ; 2v61-C18/2v5z-SAG ; 2vrl-MBN/2v60-C17 ; 2vrm-PYJ/2xfp-ISN ; 2vz2-MFG/2xfq-RAS ; 2xcg-FA8/2xfu-FA8 ; 2xfn-XCG/3po7-ZON ; 2xfo-3PL/3zyx-MBT ; 4a7a-RGZ/4a79-P1B	38	19

Table S1: *PDBid of the subsets of protein structures evaluated.* All these structures were obtained from the Protein Data Bank database.
